# Curcumin Induces Pyroptosis-Associated Molecular Changes in Osteosarcoma Cells Correlating with the ROS/NLRP3/CASPASE-1/GSDMD Axis with Concomitant PI3K/AKT Suppression and Apoptosis Activation

**DOI:** 10.3390/nu18111831

**Published:** 2026-06-05

**Authors:** Keqing Yuan, Xingyu Zhao, Jiayi Guo, Yue Lu, Yufei Cui, Wei Zhang, Wenhe Zhu

**Affiliations:** 1 Jilin Provincial Technology Collaborative Innovation Center of Hypobaric Hypoxia, School of Basic Medical Sciences, Jilin Medical University, Jilin 132013, China; 2 Graduate School of Medicine, Tohoku University, Sendai 980-8575, Japan; 3 Department of Biochemistry and Molecular Biology, School of Basic Medical Sciences, Jilin Medical University, Jilin 132013, China

**Keywords:** curcumin, osteosarcoma, pyroptosis, NLRP3 inflammasome, GSDMD

## Abstract

Curcumin, a natural polyphenolic compound derived from turmeric, exhibits broad-spectrum anticancer activities, but its ability to induce pyroptosis in osteosarcoma remains unknown. Osteosarcoma is the most common primary malignant bone tumor in children and adolescents, and novel therapeutic strategies are urgently needed to overcome osteosarcoma chemoresistance. **Aim:** This study aimed to investigate whether curcumin induces pyroptosis-associated molecular changes in human osteosarcoma cells and to explore the underlying molecular mechanisms, focusing on the ROS/NLRP3/CASPASE-1/GSDMD axis and the PI3K/AKT signaling pathway. **Methods:** Human osteosarcoma U2OS and MG63 cells were treated with curcumin (20–40 μmol·L^−1^ for 24 h). Cell viability was assessed by CCK-8 assay. Pyroptotic morphology was observed by scanning electron microscopy. Lactate dehydrogenase (LDH) release was measured colorimetrically, and IL-1β/IL-18 secretion was quantified by ELISA. Mitochondrial membrane potential (ΔΨm) and intracellular reactive oxygen species (ROS) levels were analyzed by flow cytometry. Protein expression levels of NLRP3, cleaved CASPASE-1, GSDMD-N, PI3K, AKT, p-AKT, Bax, Bcl-2 and cleaved CASPASE-3 were detected by Western blotting. Pharmacological validation was performed using the pan-caspase inhibitor Z-VAD-FMK. **Results:** Curcumin significantly inhibited the proliferation of U2OS and MG63 cells in a dose- and time-dependent manner. Scanning electron microscopy revealed characteristic pyroptotic features including cell swelling, membrane pore formation, and rupture. Curcumin treatment markedly increased LDH release and elevated IL-1β/IL-18 secretion. Mechanistically, curcumin induced mitochondrial membrane depolarization and ROS accumulation, upregulated NLRP3, cleaved CASPASE-1, and GSDMD-N expression, and concomitantly reduced PI3K/AKT pathway activity. Additionally, curcumin upregulated pro-apoptotic Bax, downregulated anti-apoptotic Bcl-2, and activated cleaved CASPASE-3. The pan-caspase inhibitor Z-VAD-FMK partially reversed curcumin-induced cytotoxicity, confirming that caspase-dependent apoptosis contributes to the overall anticancer effect. **Conclusions:** This study provides evidence that curcumin induces both apoptosis and pyroptosis-associated molecular changes in human osteosarcoma cells. The pyroptotic effect involves the ROS/NLRP3/CASPASE-1/GSDMD axis, accompanied by PI3K/AKT suppression, while caspase-dependent apoptosis also plays an important role. These findings uncover a previously unreported mechanism of curcumin’s anti-osteosarcoma activity and suggest that targeting multiple cell death pathways may represent a promising strategy to overcome apoptosis resistance in osteosarcoma.

## 1. Introduction

Curcumin, a polyphenolic compound isolated from the rhizome of *Curcuma longa*, has attracted extensive attention in cancer research for its unique structural properties and pleiotropic bioactivities [1]. Its chemical structure is characterized by a β-diketone (diferuloylmethane) core, with two methoxyphenyl rings connected by a seven-carbon linker containing an α,β-unsaturated carbonyl moiety, a configuration that endows curcumin with distinctive electron-donating and chelating properties underlying its potent antioxidant, anti-inflammatory, and broad-spectrum anticancer activities [2]. Extensive evidence has demonstrated that curcumin interferes with multiple key oncogenic signaling pathways, including NF-κB, MAPK, PI3K/Akt/mTOR, JAK/STAT, and Wnt/β-catenin, thereby inhibiting tumor cell proliferation, survival, invasion, and angiogenesis [3,4,5]. Furthermore, curcumin has been shown to enhance the efficacy of conventional chemotherapy and radiotherapy while simultaneously protecting normal tissues from toxicity, further highlighting its potential as an adjunctive anticancer agent [6,7].

Osteosarcoma is the most common primary malignant bone tumor in children and adolescents, and the 5-year survival rate for patients with metastatic or recurrent disease remains below 30% [8,9]. Extensive evidence has demonstrated that curcumin inhibits proliferation, induces apoptosis, suppresses migration and invasion, and enhances chemosensitivity in osteosarcoma cell lines, including MG63, U2OS, HOS, and 143B [9,10]. Mechanistic studies have revealed that curcumin regulates multiple signaling pathways in osteosarcoma, including MAPK/JNK, PI3K/AKT, Wnt/β-catenin, Notch, and STAT3; additionally, some studies have implicated autophagy and microRNA (miRNA) networks in its antitumor effects [9,11,12]. However, the majority of these studies have focused on apoptosis and autophagy, while the role of pyroptosis in curcumin-treated osteosarcoma remains almost entirely unexplored [9,10].

In osteosarcoma, inactivation of the apoptotic machinery—including BCL-2 overexpression, CASPASE-3 mutation, and TP53 inactivation—represents a major mechanism underlying chemoresistance and clinical treatment failure [13]. In contrast, pyroptosis relies on a distinct molecular apparatus, namely the NLRP3 inflammasome, CASPASE-1/4/5/11, and gasdermin D (GSDMD), whose activation is independent of core apoptotic components [14]. Consequently, inducing pyroptosis may bypass apoptosis resistance and provide an alternative route to eliminate drug-resistant osteosarcoma cells [15]. Furthermore, pyroptosis is characterized by the release of the pro-inflammatory cytokines interleukin (IL)-1β and IL-18, which possess direct antitumor effects and can reshape the tumor immune microenvironment [15]. At present, curcumin has already been shown to trigger pyroptosis in other solid tumors, including hepatocellular carcinoma and breast cancer [16,17,18]. Therefore, exploring curcumin-induced pyroptosis in osteosarcoma fills the research gap and may overcome the limitations of apoptosis-focused therapies [14].

Based on the above evidence, we hypothesized that curcumin induces pyroptosis in human osteosarcoma cells via inhibiting the PI3K/AKT pathway, which was verified by CCK-8 assay, immunofluorescence staining, ELISA and flow cytometry.

## 2. Materials and Methods

### 2.1. Cell Culture and Curcumin Treatment

The human osteosarcoma cell lines U2OS and MG63 were obtained from Procell Life Science & Technology Co., Ltd. (Wuhan, China) and Servicebio Technology Co., Ltd. (Wuhan, China), respectively. Cells were cultured in Dulbecco’s Modified Eagle Medium (DMEM, GIBCO, Grand Island, NY, USA) supplemented with 10% fetal bovine serum (FBS, Abcell, Shanghai, China) and 100 U/mL penicillin-streptomycin at 37 °C in a humidified atmosphere containing 5% CO_2_. U2OS and MG63 cells were authenticated by STR profiling and tested negative for mycoplasma contamination.

Curcumin (purity ≥ 99.85%, Sigma-Aldrich, St. Louis, MO, USA) was dissolved in dimethyl sulfoxide (DMSO, Biosharp, Hefei, China) to prepare a 200 mM stock solution, which was stored at −80 °C protected from light. For experiments, the stock solution was freshly diluted with culture medium to the indicated concentrations (20, 40, 60, and 80 μmol·L^−1^), with the final DMSO concentration not exceeding 0.1% (*v*/*v*) in all treatment groups. All experiments were performed with at least three independent biological replicates (*n* = 3), unless otherwise specified.

The molecular characteristics of U2OS and MG63 cells were retrieved from the literature and public databases. U2OS cells harbor wild-type TP53 with functional p53 activity [19,20], whereas MG63 cells are p53-null due to a homozygous deletion/mutation [20,21]. Both cell lines express wild-type CASP3 [14]. BCL2 expression is relatively low in U2OS compared to MG63 [19]. These features are consistent with their respective origins and are relevant to the interpretation of apoptosis and pyroptosis induction.

This study did not involve human subjects, animal experiments, or clinical samples. All cell lines used in this study were commercially available immortalized cell lines. According to the ethical guidelines of Jilin Medical University, research using established commercial cell lines is exempt from institutional ethical review. Therefore, no ethical approval was required for this work.

### 2.2. Cell Viability Assay (CCK-8)

Cell viability was assessed using the Cell Counting Kit-8 (CCK-8, Dojindo, Kumamoto, Japan) assay [22]. U2OS and MG63 cells were seeded in 96-well plates at 1 × 10^4^ cells per well and cultured for 24 h, followed by treatment with curcumin (0, 20, 40, 60, and 80 μmol·L^−1^) for 24 h, 48 h, or 72 h. CCK-8 solution (10 μL) was added to each well and incubated for 1 h at 37 °C. The absorbance was measured at 450 nm using a microplate reader (BIO-RAD, Hercules, CA, USA). The half-maximal inhibitory concentration (IC_50_) was calculated using GraphPad Prism 9.0.

### 2.3. Morphological Observation

#### 2.3.1. Phase-Contrast Microscopy

For morphological assessment, cells were seeded in 6-well plates at a density of 1 × 10^5^ cells per well and cultured for 24 h. After treatment with curcumin (0, 20, and 40 μmol·L^−1^) for 24 h, cell morphology was observed and photographed using an inverted phase-contrast microscope (Olympus, Tokyo, Japan), and images were processed with Olympus cellSens Standard 1.18.

#### 2.3.2. Scanning Electron Microscopy (SEM)

Scanning electron microscopy was employed to visualize detailed ultrastructural changes. Cells were seeded on glass coverslips in 6-well plates at 1 × 10^4^ cells per well. After curcumin treatment (0, 20, and 40 μmol·L^−1^) for 24 h, cells were fixed with 2.5% glutaraldehyde for 2 h at room temperature, post-fixed with 1% osmium tetroxide, dehydrated through a graded ethanol series, critical-point dried, sputter-coated with gold, and examined under a scanning electron microscope (Hitachi, Tokyo, Japan). Image acquisition was performed using the instrument’s built-in software (Hitachi S-3400N, Japan). No additional image analysis software was used for quantification of SEM images.

### 2.4. TUNEL Staining

DNA fragmentation was detected using the TUNEL assay [23]. Cells were seeded on sterile coverslips in 24-well plates at a density of 1 × 10^4^ cells per well and cultured for 24 h. Following curcumin treatment (0, 20, and 40 μmol·L^−1^) for 24 h, cells were fixed with 4% paraformaldehyde for 15 min at room temperature and permeabilized with 0.5% Triton X-100 for 15 min. After washing with PBS, cells were incubated with TdT reaction mixture (recombinant TdT enzyme: FITC-12-dUTP Labeling Mix: Equilibration Buffer = 1:5:50) for 1 h at 37 °C in the dark. Nuclei were counterstained with DAPI for 8 min. Coverslips were mounted with anti-fade mounting medium and observed under a laser scanning confocal microscope (Olympus, Japan), and confocal images were acquired using Olympus cellSens Standard 1.18.

### 2.5. Annexin V-FITC/PI Double Staining

Cell death was quantified using Annexin V-FITC/PI double staining [24]. After curcumin treatment (0, 20, and 40 μmol·L^−1^) for 24 h, cells were harvested, washed twice with cold PBS, and resuspended in 1× binding buffer at a concentration of 1–5 × 10^6^ cells/mL. Then, 100 μL of cell suspension was mixed with 5 μL of Annexin V-FITC and 5 μL of PI, incubated for 8–10 min at room temperature in the dark, and analyzed using a C6 flow cytometer (BD Biosciences, Franklin Lakes, NJ, USA) within 1 h. Data were analyzed using BD FACSuite software version 1.2.5 (BD Biosciences, USA).

Flow cytometry gating strategy: For each sample, at least 10,000 events were acquired. A forward scatter (FSC) vs. side scatter (SSC) gate was first applied to exclude debris and cell doublets. Single cells were then gated using FSC-height (FSC-H) vs. FSC-area (FSC-A). From the single-cell gate, the populations of Annexin V-FITC positive (FITC channel) and propidium iodide positive (PE channel) were analyzed. Data were acquired on a BD Accuri C6 flow cytometer and analyzed using BD FACSuite software (version 1.2.5).

### 2.6. Lactate Dehydrogenase (LDH) Release Assay

LDH release was measured using a colorimetric assay [25]. Cells were seeded in 96-well plates and treated with curcumin (0, 20, and 40 μmol·L^−1^) for 24 h. Culture supernatants (80 μL) were transferred to new 96-well plates and mixed with 80 μL of LDH working solution. After incubation at 37 °C for 30 min in the dark, the absorbance was measured at 450 nm using a microplate reader (BIO-RAD, Model 680, USA).

### 2.7. Enzyme-Linked Immunosorbent Assay (ELISA) for IL-1β and IL-18

IL-1β and IL-18 levels in culture supernatants were quantified using commercial ELISA kits (ZEN-BIOSCIENCE, Chengdu, China) following the manufacturer’s instructions [26]. These cytokines are hallmarks of pyroptosis, as they are cleaved and secreted upon inflammasome activation [23]. After curcumin treatment (0, 20, and 40 μmol·L^−1^) for 24 h, cell culture supernatants were collected and centrifuged at 4000 rpm for 20 min at 4 °C to remove cellular debris. The levels of IL-1β and IL-18 were determined using ELISA kits (ZEN-BIOSCIENCE, China) following the manufacturer’s protocol. Absorbance was measured at 450 nm using a microplate reader (BIO-RAD, Model 680, USA).

### 2.8. Mitochondrial Membrane Potential (ΔΨm) Assay

Mitochondrial membrane potential was assessed using the JC-1 probe [27]. After curcumin treatment (0, 20, and 40 μmol·L^−1^) for 24 h, cells were harvested, washed with PBS, and incubated with JC-1 staining solution (Beyotime, Shanghai, China) at 37 °C for 20 min. Cells were then washed twice with JC-1 staining buffer, resuspended, and analyzed by flow cytometry (BD C6, USA). Data were analyzed using BD FACSuite software version 1.2.5.

Flow cytometry gating strategy: A minimum of 10,000 events per sample were recorded. Debris and cell doublets were excluded using an FSC vs. SSC gate, followed by single-cell gating based on FSC-H vs. FSC-A. From the single-cell population, JC-1 fluorescence was measured in the FL1 (green, JC-1 monomer, 530 nm) and FL2 (orange-red, JC-1 aggregates, 585 nm) channels. The ΔΨm was expressed as the ratio of FL2 (aggregates) to FL1 (monomers).

### 2.9. Reactive Oxygen Species (ROS) Detection

Intracellular ROS levels were measured using the DCFH-DA probe [28]. Following curcumin treatment (0, 20, and 40 μmol·L^−1^) for 24 h, cells were harvested and washed with PBS. Cells were then incubated with DCFH-DA (10 μmol·L^−1^, Servicebio, China) in serum-free medium for 30 min at 37 °C in the dark. After washing with PBS three times, fluorescence intensity was immediately measured by flow cytometry (BD BIOSCIENCES, Franklin Lakes, NJ, USA). Data were analyzed using BD FACSuite software version 1.2.5.

Flow cytometry gating strategy: At least 10,000 events per sample were acquired. An FSC vs. SSC gate was used to remove debris and cell aggregates, followed by single-cell discrimination using FSC-H vs. FSC-A. The mean fluorescence intensity (MFI) of DCF (excitation 488 nm, emission 525 nm) was measured in the FL1 channel from the single-cell gate.

### 2.10. Western Blotting

#### 2.10.1. Protein Extraction and Quantification

Total cellular proteins were extracted using RIPA lysis buffer (Beyotime, China) supplemented with 1% phenylmethylsulfonyl fluoride (PMSF, Beyotime, China) at a ratio of 100:1. After curcumin treatment, cells were lysed on ice for 30 min, scraped, and sonicated. Lysates were centrifuged at 12,000 rpm for 12 min at 4 °C, and the supernatants were collected. Protein concentrations were determined using the bicinchoninic acid (BCA) method. The BCA assay is a colorimetric method based on the reduction of Cu^2+^ to Cu^+^ by proteins in an alkaline medium, followed by chelation with bicinchoninic acid to produce a purple chromophore that absorbs at 562 nm [29].

#### 2.10.2. SDS-PAGE and Immunoblotting

Equal amounts of protein (30–50 μg per lane) were separated by 12% sodium dodecyl sulfate-polyacrylamide gel electrophoresis (SDS-PAGE) and transferred onto polyvinylidene difluoride (PVDF) membranes (LABSELECT, Hefei, China). Membranes were blocked with 5% non-fat milk in PBST (PBS containing 0.1% Tween-20) for 1.5 h at room temperature and then incubated overnight at 4 °C with primary antibodies against GSDMD-N (1:1000, Abclonal, Wuhan, China), NLRP3 (1:1000, ZEN-BIOSCIENCE, China), Cleaved CASPASE-1 (1:1000, ZEN-BIOSCIENCE, China), PI3K (1:1000, ZEN-BIOSCIENCE, China), AKT (1:1000, ZEN-BIOSCIENCE, China), p-AKT (1:1000, ZEN-BIOSCIENCE, China), Bax (1:1000, ZEN-BIOSCIENCE, China), Bcl-2 (1:1000, ZEN-BIOSCIENCE, China), Cleaved CASPASE-3 (1:1000, ZEN-BIOSCIENCE, China) and GAPDH (1:1000, Santa Cruz Biotechnology, Dallas, TX, USA). After washing with PBST, membranes were incubated with HRP-conjugated goat anti-rabbit IgG secondary antibody (1:5000, Servicebio, China) for 2 h at room temperature. Protein bands were visualized using an enhanced chemiluminescence (ECL) kit (Beyotime, China) and detected with a gel imaging system (GENE, Cambridge, UK). Band intensities were quantified using Image J software version 1.53t (National Institutes of Health, Bethesda, MD, USA). Target protein expression levels were normalized to GAPDH, and the fold-change relative to the control group was calculated as the mean ± SD of three independent biological replicates.

### 2.11. Pharmacological Inhibitor Treatment

To validate the contribution of caspase-dependent cell death, cells were pre-treated with 20 μmol·L^−1^ pan-caspase inhibitor Z-VAD-FMK (Selleck, Houston, TX, USA) for 2 h, followed by co-treatment with 20 μmol·L^−1^ or 40 μmol·L^−1^ curcumin for 24 h. Cell viability was then determined using the CCK-8 assay as described above.

### 2.12. Statistical Analysis

All experiments were performed independently at least three times. Data are presented as mean ± standard deviation (SD). Statistical analyses were conducted using GraphPad Prism 9.0 software (GraphPad Software, Boston, MA, USA). Differences among multiple groups were evaluated using one-way analysis of variance (ANOVA), followed by Tukey’s honestly significant difference (HSD) post hoc test for pairwise comparisons. Exact *p* values are reported where applicable; *p* < 0.05 was considered statistically significant, and *p* < 0.01 indicated high statistical significance.

## 3. Results

### 3.1. Effect of Curcumin on the Proliferation of U2OS and MG63 Cells

**Figure 1:**
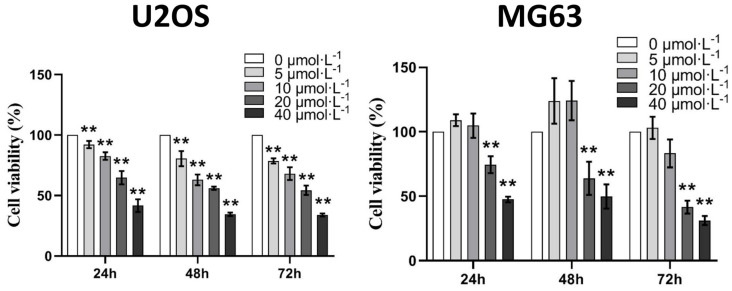
U2OS cell and MG63 cell proliferation activity in various groups. Curcumin inhibits the viability of U2OS and MG63 cells. Cells were treated with indicated concentrations of curcumin for 24, 48, or 72 h. Viability was measured by CCK-8 assay. Data are presented as mean ± SD (*n* = 3). **: *p* < 0.01 vs. control group.

CCK-8 assay results showed that curcumin (Cur) significantly inhibited the proliferation of human osteosarcoma U2OS and MG63 cells in a concentration- and time-dependent manner (Figure 1). Low concentration of Cur (5 μmol·L^−1^) had no obvious inhibitory effect on the viability of both cell lines. Within the concentration range of 10~40 μmol·L^−1^, the viability of U2OS and MG63 cells decreased significantly with the increase in Cur concentration and the extension of treatment time (24 h, 48 h, 72 h), with a highly statistically significant difference compared with the control group (*p* < 0.01). Based on these results, curcumin concentrations of 20 and 40 μmol·L^−1^ were selected for subsequent mechanistic experiments.

Consistent with the CCK-8 results, phase-contrast microscopy revealed dose-dependent morphological changes including cell shrinkage, rounding, and detachment following curcumin treatment (Figure S1), further supporting the anti-proliferative effect of curcumin.

### 3.2. Curcumin Induces Pyroptotic Morphology (SEM)

**Figure 2:**
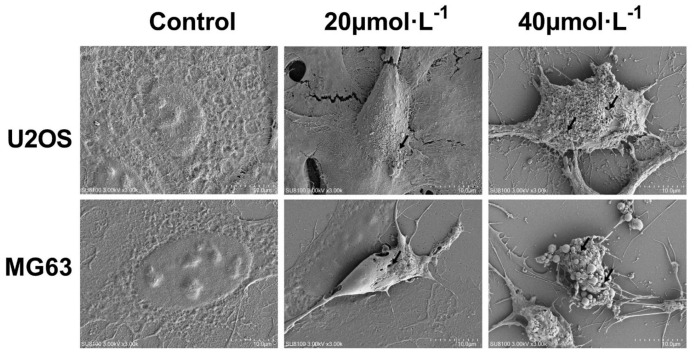
Morphological changes in cells under SEM (3k×, scale bar = 10 μm).

Figure 2 revealed the ultrastructural changes in U2OS and MG63 osteosarcoma cells after 24 h curcumin (Cur) treatment. Control cells showed intact morphology with smooth membrane and abundant filopodia. A total of 20 μmol·L^−1^ Cur impaired membrane integrity and induced pore formation (black arrows), while 40 μmol·L^−1^ Cur triggered typical pyroptotic changes including severe cell swelling, membrane rupture, vesicular protrusions and cellular content release. The cell damage was Cur concentration-dependent.

**Figure 3:**
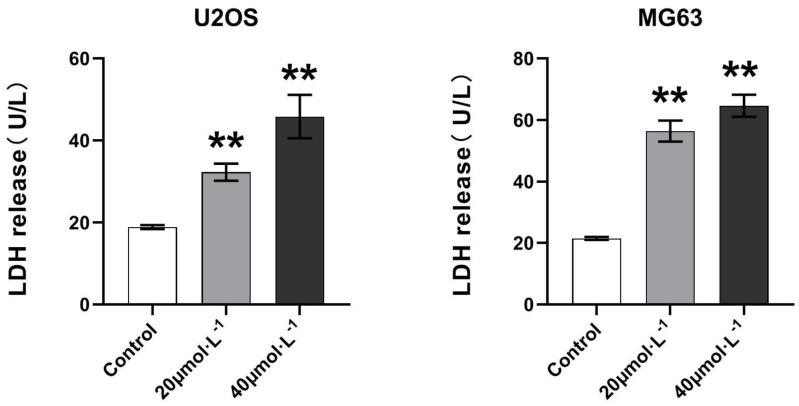
The effect of curcumin on concentration of LDH in osteosarcoma cells. *n* = 3; **: *p* < 0.01 vs. control group.

**Figure 4:**
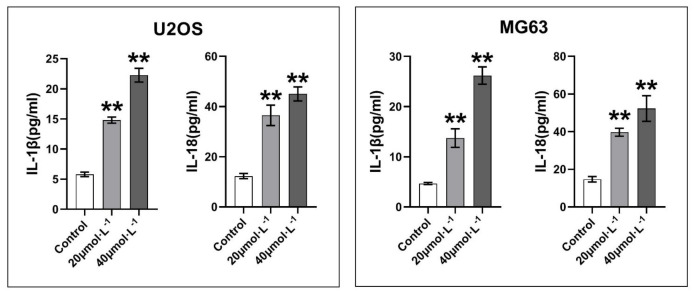
The effect of different concentrations of curcumin on release levels both of IL-1β and IL-18 in osteosarcoma cells. *n* = 3; **: *p* < 0.01 vs. control group.

To distinguish pyroptosis from apoptosis, we employed orthogonal assays: scanning electron microscopy for membrane pore formation [19] (Figure 2), LDH release and IL-1β/IL-18 secretion (Figure 3 and Figure 4) TUNEL-positive DNA fragmentation (Figure S2) was considered a non-specific downstream event, as it occurs in multiple cell death pathways.

### 3.3. Curcumin Causes LDH Release and IL-1β/IL-18 Secretion

Disruption of cell membrane integrity is one of the core characteristics that distinguish pyroptosis from apoptosis. ELISA results are presented in Figure 3 and Figure 4.

As shown in Figure 3, compared with the control group, 24 h treatment with 20 μmol·L^−1^ and 40 μmol·L^−1^ curcumin significantly increased LDH levels in the supernatant of both U2OS and MG63 cells in a concentration-dependent manner, with a highly statistically significant difference (*p* < 0.01). These results indicated that curcumin markedly impaired the membrane integrity of osteosarcoma cells and induced membrane rupture, which was consistent with the core morphological features of pyroptosis.

In Figure 4, curcumin treatment significantly upregulated the secretion of IL-1β and IL-18 in U2OS and MG63 cells in a clear concentration-dependent manner. Compared with the control group, the levels of IL-1β and IL-18 in both cell lines were significantly elevated after 20 μmol·L^−1^ and 40 μmol·L^−1^ curcumin intervention (*p* < 0.01), which confirmed that curcumin could activate pyroptosis-related inflammatory response and trigger pyroptosis in osteosarcoma cells.

Flow cytometric analysis using Annexin V-FITC/PI double staining further demonstrated that curcumin (20 and 40 μmol·L^−1^, 24 h) dose-dependently increased the proportion of Annexin V^+^/PI^+^ cells in U2OS and MG63 cultures (*p* < 0.01, Figure S3), indicating a marked increase in total cell death. Consistent with the subsequent pyroptosis-specific assays, this result supports the conclusion that curcumin effectively eliminates osteosarcoma cells.

### 3.4. Curcumin Induces Mitochondrial Dysfunction and ROS Production

**Figure 5:**
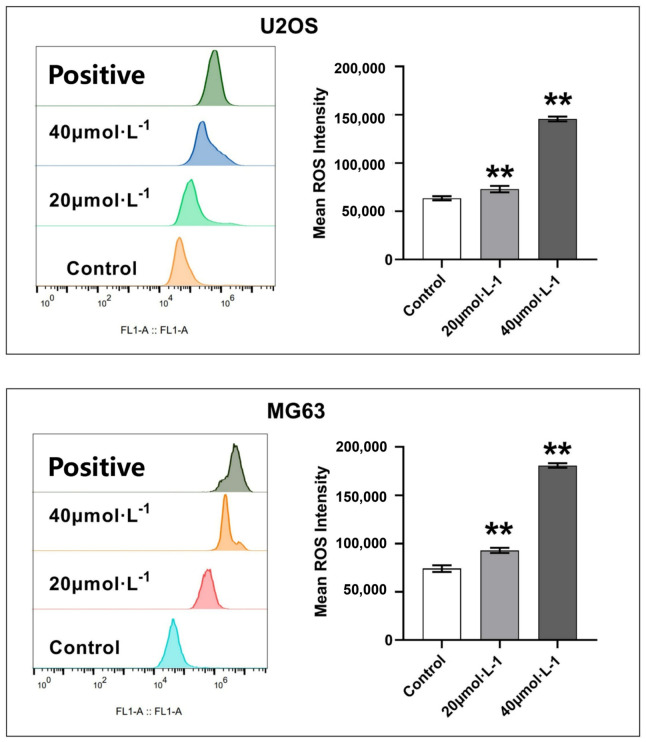
Effects of different concentrations of curcumin on intracellular ROS levels in osteosarcoma cells detected by flow cytometry. **: *p* < 0.01 vs. control group.

Flow cytometry results revealed (Figure 5), compared with the control group, that 24 h treatment with 20 μmol·L^−1^ and 40 μmol·L^−1^ curcumin induced a concentration-dependent right shift in the fluorescence peak in both cell lines. Quantitative results of mean ROS fluorescence intensity showed that intracellular ROS levels were significantly elevated in curcumin-treated groups, with a more prominent increase in the 40 μmol·L^−1^ group, and the differences were highly statistically significant (*p* < 0.01). These results indicated that curcumin could induce intracellular ROS accumulation in osteosarcoma cells in a concentration-dependent manner.

**Figure 6:**
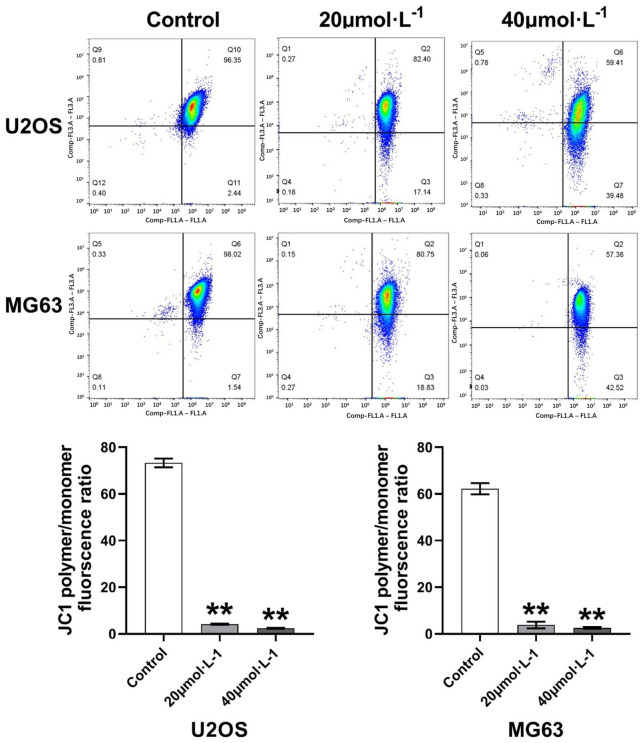
Effect of different concentrations of curcumin on mitochondrial membrane potential (ΔΨm) in osteosarcoma cells. JC-1 fluorescence: red/orange aggregates (FL2 channel, 585 nm) indicate high ΔΨm, while green monomers (FL1 channel, 530 nm) indicate depolarization. The ratio of FL2/FL1 is presented in the bar graph. *n* = 3; **: *p* < 0.01 vs. control.

In the control group, cells maintained normal mitochondrial function, with JC-1 mainly existing as orange–red fluorescent aggregates, over 97% of cells distributed in the Q2 quadrant, and a high JC-1 polymer/monomer fluorescence ratio. After 24 h treatment with 20 μmol·L^−1^ and 40 μmol·L^−1^ curcumin, significant mitochondrial membrane depolarization was observed in both cell lines: JC-1 aggregates decreased while green fluorescent monomers increased markedly, and the proportion of cells decreased in the Q2 quadrant and increased in the Q3 quadrant in a concentration-dependent manner. Quantitative results showed that the JC-1 polymer/monomer fluorescence ratio in curcumin-treated groups was significantly lower than that in the control group (*p* < 0.01) in a concentration-dependent manner. These results indicated that curcumin could significantly impair mitochondrial function and induce mitochondrial membrane depolarization in osteosarcoma cells (Figure 6).

### 3.5. Curcumin Activates the NLRP3/CASPASE-1/GSDMD Pyroptosis Pathway (Western Blot)

**Figure 7:**
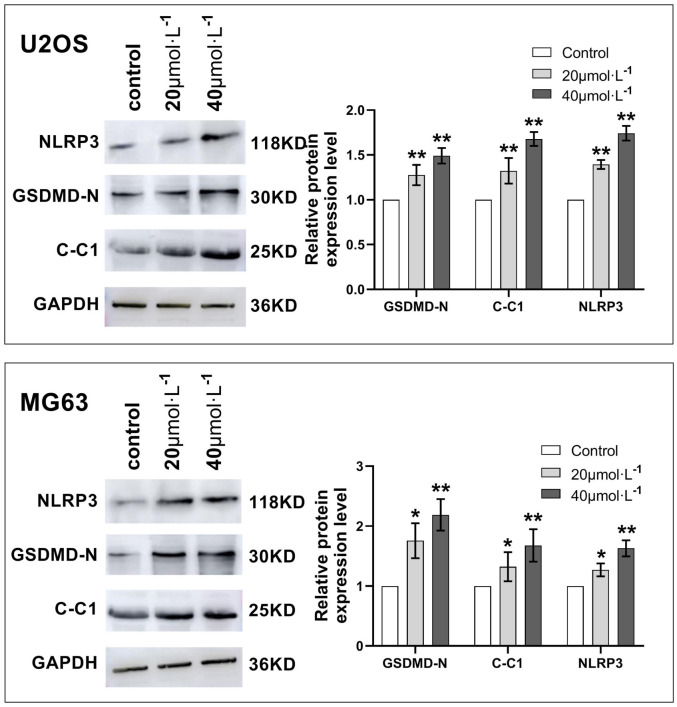
The effect of different concentrations of curcumin on the levels of pyroptosis-related proteins in osteosarcoma cells. Representative Western blots and quantitative analysis of NLRP3, GSDMD-N and cleaved CASPASE-1 (C-C1) protein expression in U2OS and MG63 cells. Data are presented as mean ± SD of three independent biological replicates. *: *p* < 0.05, **: *p* < 0.01 vs. control group.

The results of Western blotting showed (Figure 7), compared with the control group, that 24 h intervention with 20 μmol·L^−1^ and 40 μmol·L^−1^ curcumin significantly upregulated the protein levels of NLRP3 inflammasome, cleaved CASPASE-1, and the pyroptosis executive active fragment GSDMD-N in both cell lines in a concentration-dependent manner, with statistically significant differences. In U2OS cells, all three proteins were significantly upregulated at both 20 and 40 μmol·L^−1^ (*p* < 0.01). In MG63 cells, 20 μmol·L^−1^ curcumin induced significant upregulation of GSDMD-N (*p* < 0.05), cleaved CASPASE-1 (*p* < 0.05) and NLRP3 (*p* < 0.05), while 40 μmol·L^−1^ curcumin caused more pronounced upregulation of all three proteins (*p* < 0.01).

### 3.6. Curcumin Inhibits the PI3K/AKT Pathway (Western Blot)

**Figure 8:**
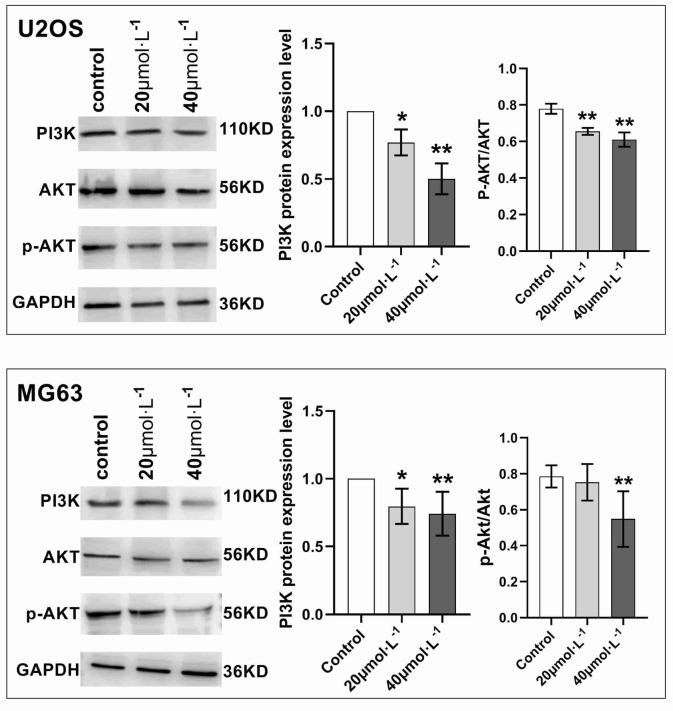
The effect of different concentrations of curcumin on the levels of PI3K/AKT signaling pathway-related proteins in osteosarcoma cells. *n* = 3; *: *p* < 0.05, **: *p* < 0.01 vs. control group.

Western blotting was performed to detect the expression of key proteins in the PI3K/AKT signaling pathway in U2OS and MG63 osteosarcoma cells after curcumin treatment (Figure 8). Compared with the control group, 24 h intervention with 20 μmol·L^−1^ and 40 μmol·L^−1^ curcumin significantly downregulated the protein expression of PI3K in both cell lines in a concentration-dependent manner. There was no significant difference in the expression of total AKT (AKT) among all groups, while the expression level of phosphorylated AKT (p-AKT) and the p-AKT/AKT ratio were significantly decreased in a concentration-dependent manner, with statistically significant differences compared with the control group (*: *p* < 0.05, **: *p* < 0.01). These results indicated that curcumin could markedly inhibit the activation of the PI3K/AKT signaling pathway in osteosarcoma cells.

### 3.7. Pharmacological Validation of Caspase-Dependent Cell Death and Apoptosis-Related Protein Expression

**Figure 9:**
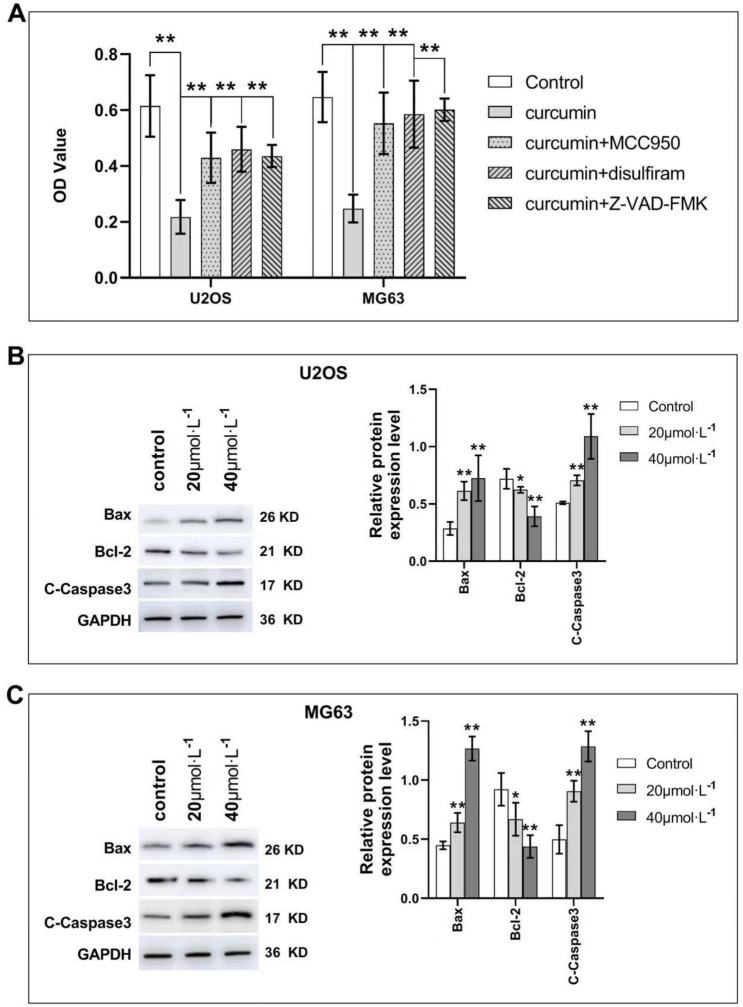
Pharmacological validation of cell death pathways and apoptosis-related protein expression. (**A**) Effect of Z-VAD-FMK on curcumin-induced cytotoxicity in U2OS and MG63 cells. Cells were pre-treated with 20 μmol·L^−1^ Z-VAD-FMK for 2 h, followed by co-treatment with 20 μmol·L^−1^ or 40 μmol·L^−1^ curcumin for 24 h. Cell viability was measured by CCK-8 assay. (**B**) Western blot analysis of Bax, Bcl-2 and cleaved CASPASE-3 expression in U2OS cells. (**C**) Western blot analysis of Bax, Bcl-2 and cleaved CASPASE-3 expression in MG63 cells. Data are presented as mean ± SD (*n* = 3). *: *p* < 0.05, **: *p* < 0.01 vs. control group.

To distinguish the contributions of different forms of regulated cell death to curcumin-induced cytotoxicity, we performed co-treatment experiments with the pan-caspase inhibitor Z-VAD-FMK. As shown in Figure 9A, pre-treatment with Z-VAD-FMK significantly reversed the curcumin-induced decrease in cell viability in both U2OS and MG63 cells (*p* < 0.01), indicating that caspase-dependent cell death plays an important role in the anticancer effect of curcumin.

To further confirm the involvement of apoptosis, we detected the expression of key apoptosis-related proteins by Western blotting. As shown in Figure 9B,C, curcumin treatment dose-dependently upregulated the expression of pro-apoptotic Bax and cleaved CASPASE-3, while downregulating the expression of anti-apoptotic Bcl-2 in both U2OS and MG63 cells (*p* < 0.05 or *p* < 0.01). These results collectively demonstrate that curcumin induces both caspase-dependent apoptosis and pyroptosis in osteosarcoma cells.

## 4. Discussion

In this study, we provide novel evidence that curcumin induces both caspase-dependent apoptosis- and pyroptosis-associated molecular changes in human osteosarcoma U2OS and MG63 cells, supported by morphological (SEM: cell swelling, membrane pores, rupture), functional (LDH release, IL-1β/IL-18 secretion), and molecular (upregulation of NLRP3, cleaved CASPASE-1, and GSDMD-N) [30] evidence for pyroptosis, as well as upregulation of Bax, downregulation of Bcl-2, activation of cleaved CASPASE-3, and partial reversal of cytotoxicity by the pan-caspase inhibitor Z-VAD-FMK for apoptosis, all consistent with established criteria for regulated cell death [31,32].

A TUNEL assay showed dose-dependent DNA fragmentation (Figure S2), a non-specific feature that can occur during various cell death pathways including pyroptosis and apoptosis [33,34]. Therefore, the identification of pyroptosis was based on orthogonal assays specific to pyroptosis: (i) the above pyroptotic features are morphologically distinct from apoptotic membrane blebbing and apoptotic bodies [35]; (ii) LDH release and IL-1β/IL-18 secretion are not hallmarks of apoptosis [32]; (iii) the upregulation of Bax/Bcl-2 ratio and activation of cleaved CASPASE-3 are specific markers of the intrinsic apoptotic pathway; and (iv) the partial reversal of cytotoxicity by Z-VAD-FMK confirms the contribution of caspase-dependent cell death. While Z-VAD-FMK inhibits both apoptotic caspases (CASPASE-3/7/9) and inflammatory caspases (CASPASE-1) involved in canonical pyroptosis, our combined molecular and functional data strongly support that both apoptosis and pyroptosis contribute to curcumin-induced cell death in osteosarcoma cells.

Notably, in this process, the decrease in mitochondrial membrane potential (Figure 6) occurred concurrently with the ROS burst (Figure 5), suggesting that the opening of the mitochondrial permeability transition pore (mPTP) may serve as a potential upstream event linking curcumin treatment to mitochondrial dysfunction, ROS generation and subsequent pyroptosis. Previous studies have shown that curcumin can directly target mitochondria and induce mPTP opening [36], which further leads to the collapse of the proton gradient, the cessation of ATP synthesis, and massive ROS production. Therefore, we propose that mitochondria serve as a key hub orchestrating the initiation of pyroptosis in curcumin-treated osteosarcoma cells.

Beyond serving as an initiating signal, our data suggest that ROS may further amplify pyroptosis through a positive feedback loop involving the PI3K/AKT pathway. We find that curcumin treatment simultaneously inhibited PI3K/AKT activity (Figure 8) and elevated ROS levels (Figure 5), suggesting an interaction between the two. Two mutually regulatory mechanisms have been reported in the literature: on the one hand, ROS can suppress the PI3K/AKT pathway by oxidizing and inactivating PTEN [36,37]; on the other hand, AKT can inhibit ROS production by promoting the expression of antioxidant enzymes [38]. Therefore, when AKT is inhibited, the antioxidant defense is weakened and ROS levels are further elevated. Based on our results and previous reports, we hypothesize that a positive feedback loop may exist in curcumin-treated osteosarcoma cells: curcumin-induced mitochondrial dysfunction generates an initial ROS burst, which in turn suppresses the PI3K/AKT pathway. The subsequent reduction in antioxidant capacity further elevates ROS levels, thereby potentially sustaining NLRP3 inflammasome activation. This hypothesis remains to be validated in future studies using pathway-specific agonists or inhibitors.

In the present study, curcumin treatment significantly increased the secretion of IL-1β and IL-18 (Figure 4). These two cytokines are not only characteristic markers of pyroptosis but also key mediators of immunogenic cell death [36]. During pyroptosis, dying cells release damage-associated molecular patterns (including HMGB1, ATP, and IL-1α) as well as the pro-inflammatory cytokines IL-1β and IL-18. Collectively, these signals promote the maturation and recruitment of dendritic cells, which in turn initiate adaptive T-cell responses against tumor antigens [39,40]. Osteosarcoma is considered an immunologically “cold” tumor, characterized by low T-cell infiltration, low mutation burden, and poor response to immune checkpoint inhibitors [41]. However, because the present study was conducted exclusively in human osteosarcoma cell lines without any immune components, the immunogenic potential of curcumin-induced pyroptosis remains entirely speculative at this stage. Whether such pyroptosis-associated cytokine release can effectively recruit immune cells, reverse immune evasion, or improve responses to immune checkpoint inhibitors in vivo requires direct testing in immunocompetent animal models. Therefore, we wish to emphasize that no conclusion regarding the conversion of “cold” tumors into “hot” tumors can be drawn from the current data; the immunogenic potential of curcumin-induced pyroptosis in osteosarcoma is a question that awaits future in vivo investigation.

This study provides the first evidence that curcumin induces pyroptosis-associated molecular changes in osteosarcoma cells via the ROS/NLRP3/CASPASE-1/GSDMD axis, accompanied by PI3K/AKT suppression. However, several issues need further exploration.

- While our orthogonal assays (SEM, LDH, IL-1β/IL-18, and Western blotting) collectively support pyroptosis as the primary cell death modality, we did not formally exclude a potential contribution of apoptosis; future studies using CASPASE-3 inhibitors or knockout models could clarify this point. Specific inhibitors of NLRP3 or GSDMD were not applied, but the combination of multiple orthogonal assays (SEM morphology, LDH release, IL-1β/IL-18 secretion, cleaved CASPASE-1 and GSDMD-N upregulation) provides robust evidence for pyroptosis.
- The observed associations between curcumin treatment and the molecular events described—including ROS accumulation, NLRP3 upregulation, and PI3K/AKT suppression—are correlative. Without direct interventional experiments using ROS scavengers (e.g., NAC), NLRP3 inhibitors (e.g., MCC950), or AKT activators (e.g., SC79), the causal relationships within the proposed ROS/NLRP3/CASPASE-1/GSDMD axis and the specific role of PI3K/AKT suppression remain to be definitively established. Such functional validation studies are essential next steps and are planned for future investigations.
- The present findings are limited to in vitro models. In vivo validation in immunocompetent orthotopic or xenograft osteosarcoma models will be essential to assess therapeutic relevance. Additionally, parallel experiments in normal human osteoblasts would be necessary to evaluate the tumor selectivity of curcumin-induced pyroptosis for malignant versus normal bone cells. The absence of such controls precludes conclusions regarding the therapeutic window and potential off-target toxicity.

Despite these limitations, our data robustly demonstrate that curcumin activates both the canonical pyroptosis machinery and the intrinsic apoptotic pathway in osteosarcoma cells, opening a new avenue for overcoming apoptosis resistance through multi-pathway targeting.

## 5. Conclusions

The present in vitro study demonstrates that curcumin induces both apoptosis- and pyroptosis-associated molecular changes in human osteosarcoma cells. Pyroptosis is associated with changes in the ROS/NLRP3/CASPASE-1/GSDMD axis, while apoptosis involves Bax/Bcl-2 dysregulation and CASPASE-3 activation, both of which are associated with PI3K/AKT suppression. Crucially, all findings are limited to cell lines; whether curcumin induces pyroptosis or modulates immune responses in vivo remains unknown. The observed elevations in IL-1β and IL-18 provide a hypothesis-generating rationale, but no claims regarding immunogenic conversion or antitumor immunity can be made. These findings reveal a dual cell death mechanism that warrants further in vivo investigation and validation.

## Data Availability

The original contributions of this study are included in the article. Further information is available from the corresponding author upon reasonable request.

## Supplementary Materials

The following supporting information can be downloaded at: https://www.mdpi.com/article/10.3390/nu18111831/s1, Figure S1: Morphological changes in U2OS cells and MG63 cells (×200); Figure S2: Nuclear changes in U2OS cells and MG63 cells (×400); Figure S3: Curcumin induces cell death in osteosarcoma cells detected by Annexin V-FITC/PI double staining.
