# Supplementary material for: Curcumin Induces Pyroptosis-Associated Molecular Changes in Osteosarcoma Cells Correlating with the ROS/NLRP3/CASPASE-1/GSDMD Axis with Concomitant PI3K/AKT Suppression and Apoptosis Activation
Source: Nutrients. 2026 Jun 5;18(11):1831. doi: 10.3390/nu18111831 (PMC13259424; doi:10.3390/nu18111831)
Supplement: Supplementary file 1 [file nutrients-18-01831-s001.zip › nutrients-4309372-supplementary.pdf]

Figure S1. Morphological changes of U2OS cells and MG63 cells (×200)

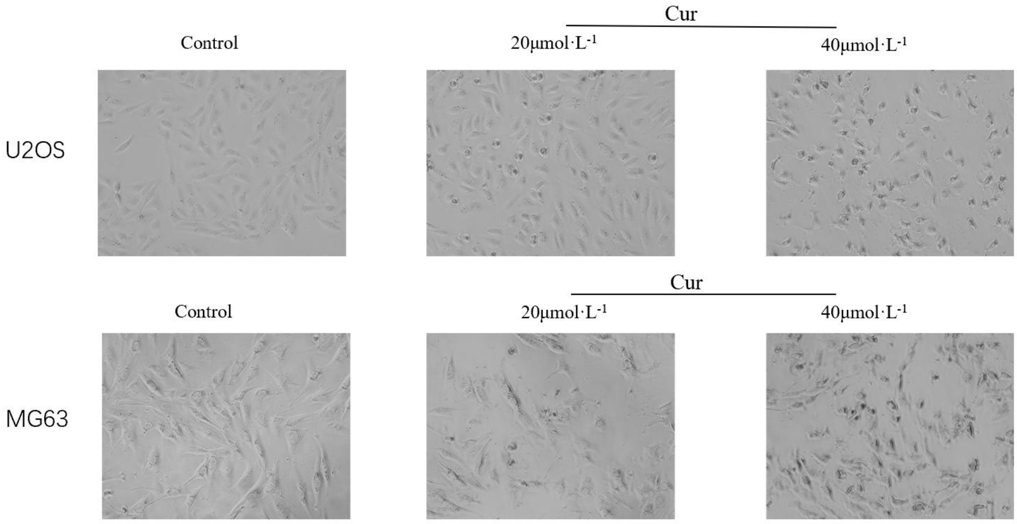

Phase-contrast microscopy (200× magnification) was used to observe the morphological changes of human osteosarcoma U2OS and MG63 cells after treatment with different concentrations of curcumin (Cur).In the control group, both U2OS and MG63 cells exhibited the typical spindle-shaped fibroblast-like morphology of osteosarcoma cells, with firm cell adhesion, good spreading status, plump cell bodies with clear outlines, tight intercellular connections, and confluent growth, without obvious cell detachment or morphological abnormalities.

After treatment with 20 $\mu mol\cdot L^{-1}$ Cur, mild morphological abnormalities were observed in both cell lines: some cells showed shrinkage, loss of the original spindle-shaped spreading structure, an initial trend of rounding, reduced adherence, widened intercellular gaps, and a decreased overall cell density compared with the control group. Notably, the morphological changes in U2OS cells were more pronounced than those in MG63 cells.

When the concentration of Cur was increased to 40 $\mu mol\cdot L^{-1}$, the morphological damage was significantly aggravated in a dose-dependent manner: the vast majority of cells displayed obvious cell shrinkage, rounding, and membrane blebbing, accompanied by severely impaired adherence, massive cell detachment from the culture substrate, complete loss of intercellular connections, a marked reduction in viable cell density in the visual field, and abundant suspended cell debris. These morphological alterations were consistent in both U2OS and MG63 osteosarcoma cell lines.

These results intuitively demonstrate the dose-dependent cytotoxicity of curcumin against osteosarcoma cells, which corroborates the anti-proliferative effect detected by MTT assay, and further supports the growth-inhibitory effect of curcumin on U2OS and MG63 cells.

Figure S2. Nuclear changes in U2OS cells and MG63 cells (×400).

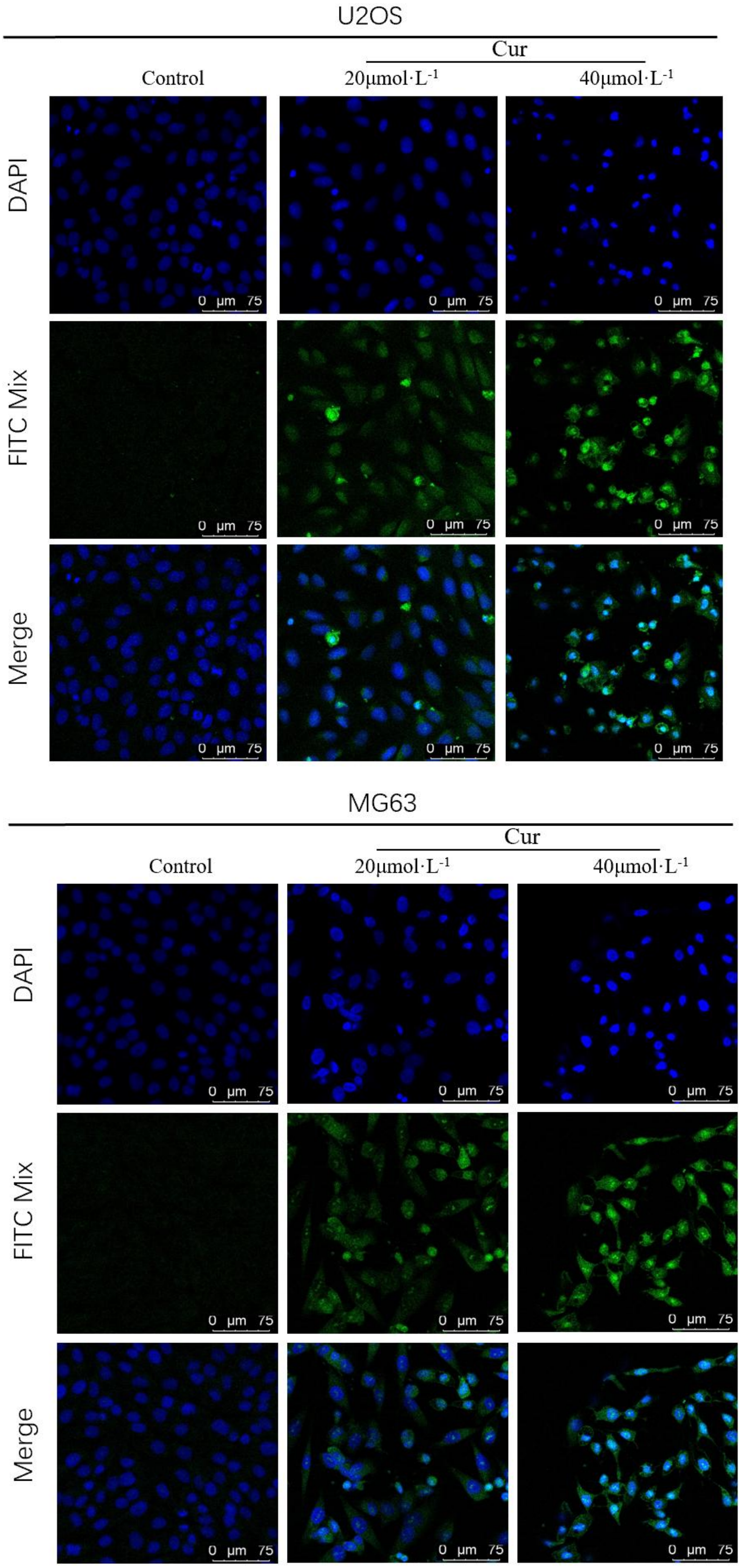

U2OS and MG63 cells were treated with curcumin (0, 20, 40 μmol·L⁻¹) for 24 h, then stained with TUNEL (green) and DAPI (blue). Representative confocal microscopy images show that

curcumin treatment increased TUNEL-positive cells in a dose-dependent manner, indicating DNA fragmentation. Scale bar = 20 μm. While DNA fragmentation is a classic feature of apoptosis, it can also occur during pyroptosis as a secondary event; the specific induction of pyroptosis was confirmed by orthogonal assays including SEM, LDH release, IL-1β/IL-18 secretion, and western blotting for cleaved caspase-1 and GSDMD-N.

Figure S3. Curcumin induces cell death in osteosarcoma cells detected by Annexin V-FITC/PI double staining. n=3.*: P < 0.05，**: P < 0.01 vs Control group.

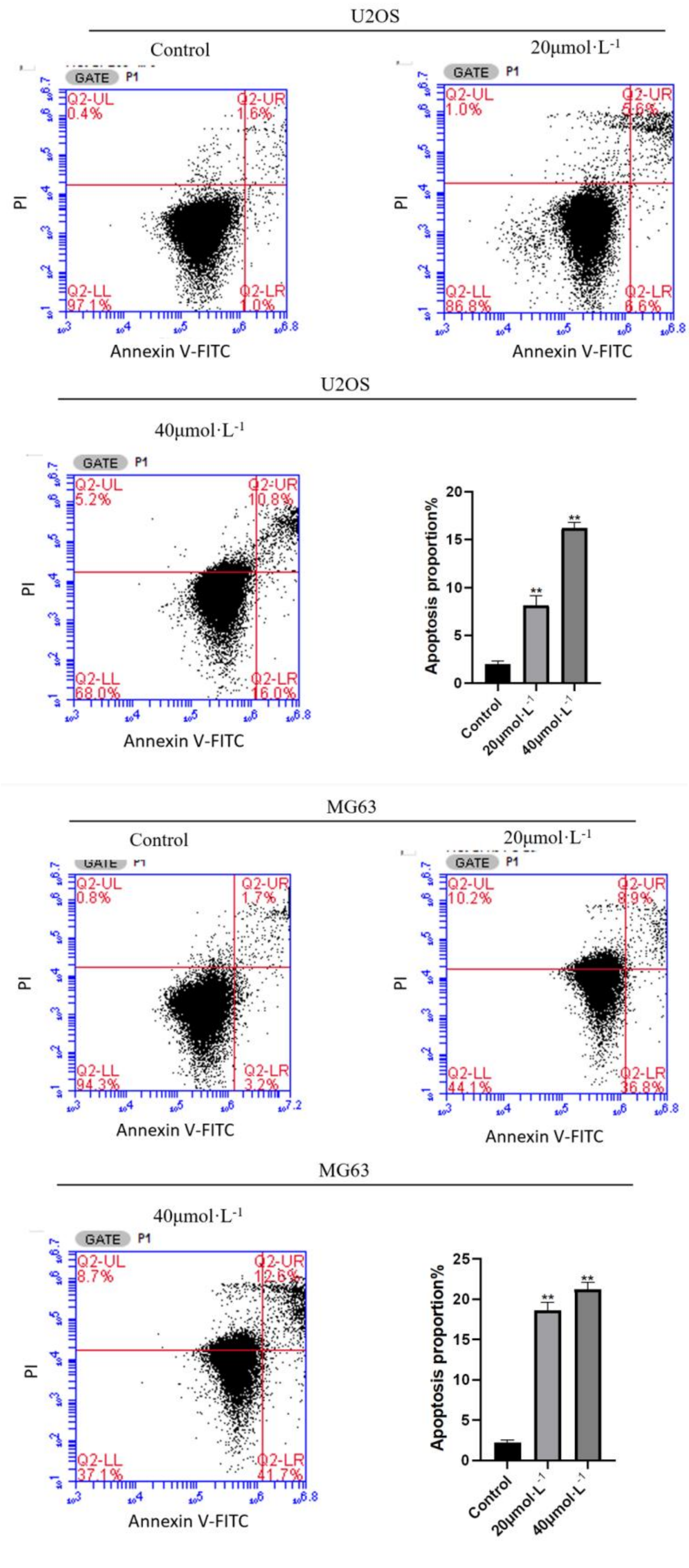

U2OS and MG63 cells were treated with the indicated concentrations of curcumin for 24 h, then stained with Annexin V-FITC and propidium iodide (PI) and analyzed by flow cytometry. The percentages of Annexin V^{+}/PI^{+} (late apoptotic/necrotic/pyroptotic) cells are shown in the corresponding quadrants. Data are presented as mean ± SD (n = 3). **P < 0.01 vs. control group. This assay detects total cell death irrespective of the specific pathway; pyroptosis was confirmed by orthogonal assays (SEM, LDH, IL-1β/IL-18, and western blotting).
